# Commentary: Efficacy of stem cell therapy for diabetic kidney disease: a systematic review and meta-analysis

**DOI:** 10.3389/fmed.2026.1700379

**Published:** 2026-02-06

**Authors:** Yunpeng Xu, Zi Yang, Xue Han, Qiuyu Chen, Aiju Su, Jian Liu

**Affiliations:** 1 The First Clinical Medical College of Lanzhou University, Lanzhou, China; 2 Information Center, The First Hospital of Lanzhou University, Lanzhou, China; 3 Gansu Province Maternity and Child-Care Hospital (Gansu Provincial Central Hospital), Lanzhou, China

**Keywords:** diabetic nephropathies, evidence-based medicine, meta-analysis, risk assessment, stem cell transplantation, systematic reviews

## Introduction

We read with great interest the recent systematic review and meta-analysis by Du et al., published in Frontiers in Medicine, entitled “Efficacy of stem cell therapy for diabetic kidney disease: A systematic review and meta-analysis” (1). This study evaluated the efficacy and safety of stem cell therapy in patients with diabetic kidney disease (DKD). We highly commend the authors for their valuable contribution to this important field. However, in the spirit of academic rigor, we would like to highlight several methodological issues that may affect the interpretation and generalizability of the findings. We respectfully offer the following points for consideration and discussion by the authors and readers.

## Study selection error

The study population of this meta-analysis should have been patients with diabetic nephropathy (DN), with intervention and control groups differing only in the intervention received. However, the authors inappropriately included the study by Gaipov et al., in which the groups were defined as “DN” vs. “type 1 diabetes (T1DM, without DN),” and both groups received stem cell infusion (2). As a result, no control group met the predefined inclusion criteria. This error introduced substantial clinical and methodological heterogeneity, rendering the interpretation of the pooled effect clinically meaningless. We recommend that this study be excluded.

## Unit-of-analysis error

In meta-analyses, when multiple intervention groups share the same control group, it is essential to avoid reusing the control data; otherwise, statistical independence is compromised, effect precision is artificially inflated, and the risk of false-positive findings increases (3). For example, the study by Packham et al. included three groups: a low-dose group (150 × 10^6^), a high-dose group (300 × 10^6^), and a placebo group (4). In this meta-analysis, however, the authors treated “low dose vs. placebo” and “high dose vs. placebo” as two independent comparisons, thereby duplicating the control group.

This error had a major impact on the pooled results. In the primary outcome Effect of SCT on eGFR, the affected study accounted for as much as 55.6% of the weight; in the outcome urine albumin/creatinine ratio, the weight reached 97.9%, seriously undermining the credibility of the findings. According to the Cochrane Handbook, this issue can be addressed by several approaches: (i) combining the intervention groups (recommended), (ii) selecting only one comparison, (iii) splitting the control group, or (iv) adopting a network meta-analysis framework. The authors did not apply any of these solutions, which represents a serious methodological flaw (3).

## Inappropriate model selection

In the methods section, the authors assessed heterogeneity solely based on the *I*^2^ statistic and used it as the criterion to choose between a fixed-effects or random-effects model. However, according to the Cochrane Handbook for Systematic Reviews of Interventions, even when *I*^2^ < 50%, a random-effects model should be preferred if there are substantial clinical or methodological differences between studies, and the rationale should be explicitly discussed (5). In this meta-analysis, the four included studies showed clear clinical heterogeneity in terms of patient populations, intervention strategies, and control groups. Nevertheless, the authors relied exclusively on the *I*^2^ statistic to apply a fixed-effects model, without considering the evident clinical heterogeneity. This represents a methodological shortcoming. We recommend reanalyzing the data using a random-effects model and providing a systematic discussion of potential sources of heterogeneity in the discussion section.

## Misuse of quality assessment tool

In the quality assessment section, the authors employed the Cochrane RoB v1 tool to evaluate risk of bias. However, according to the Cochrane Handbook for Systematic Reviews of Interventions, this tool is applicable only to randomized controlled trials (RCTs) (6). For non-randomized controlled trials (CCTs), the appropriate tool is ROBINS-I, which is specifically designed for non-randomized studies. In this review, one CCT was included, yet the authors applied the RoB v1 tool uniformly across all studies. This practice is inconsistent with methodological standards and constitutes a serious flaw. We recommend reassessing the CCT study using ROBINS-I and distinguishing the risk-of-bias evaluation according to study design in the discussion section.

## Errors in search strategy construction

In this meta-analysis, the authors searched PubMed, Embase, Web of Science, and the Cochrane Library, and provided the full search strategies in the supplementary materials. However, upon verification, we found that these search strategies contained significant errors, making it impossible to reproduce the results presented in Figure 1. The main issues were as follows: (i) incorrect use of “OR” instead of “AND” between different concepts; (ii) use of MeSH terms only for “Diabetic Nephropathies” but not for “Stem Cells”; (iii) overuse of “All Fields” in the PubMed search, rather than restricting terms to Title/Abstract as recommended in the Cochrane Handbook; (iv) missing parentheses, leading to logical priority errors; (v) spelling errors in keywords, such as “Nephropathiesl”; (vi) incorrect field tags in the Cochrane Library search, using “:t, ab, kw” instead of the correct “:ti, ab, kw”; and (vii) misuse of field tags in Web of Science, where “(Topic)” was incorrectly used instead of the proper “TS=”.

## Inconsistencies and reporting deficiencies

In Supplementary Figure S1, the authors conducted meta-analyses of HbA1c, triglycerides (TG), and glucose. However, these results were not presented in the Results section but were instead cited in the Discussion section. This approach violates reporting standards such as PRISMA for systematic reviews and meta-analyses (7). According to these guidelines, all primary analysis results should be reported in full and transparently in the Results section, rather than appearing only in supplementary materials or being mentioned solely in the discussion.

## Methodological and reporting errors in adverse event results

In the section “Adverse event reporting results,” the authors summarized adverse events in Table 3 but did not perform any quantitative analysis. Nevertheless, they incorrectly described this part as meta-analysis results (5). Furthermore, in the Discussion section, the authors stated that “Additionally, there was no significant difference in the incidence of adverse events between the two groups,” while in the Conclusion they claimed that “The meta-analysis reveals that the experimental intervention is associated with a higher overall frequency of AEs compared to the control group.” This section contains multiple problems: (i) the content represents a systematic review rather than a meta-analysis; (ii) a systematic review without quantitative synthesis cannot report “statistical significance”; and (iii) the statements in the discussion and conclusion are contradictory and logically inconsistent.

## Discussion

Although the systematic review and meta-analysis by Du et al. provides insight into the potential role of stem cell therapy in DKD, the methodological issues noted above undermine the reliability of its conclusions (1). Future studies should apply stricter inclusion criteria, use appropriate risk-of-bias tools, and adhere to Cochrane and PRISMA guidelines to improve methodological rigor and reporting standards. More robust, high-quality studies are needed to further validate the clinical potential of stem cell therapy in DKD management.
